# A Research Study of the Association between Maternal Microchimerism and Systemic Lupus Erythematosus in Adults: A Comparison between Patients and Healthy Controls Based on Single-Nucleotide Polymorphism Using Quantitative Real-Time PCR

**DOI:** 10.1371/journal.pone.0074534

**Published:** 2013-09-11

**Authors:** Anna Maria Jonsson Kanold, Elisabet Svenungsson, Iva Gunnarsson, Cecilia Götherström, Leonid Padyukov, Nikos Papadogiannakis, Mehmet Uzunel, Magnus Westgren

**Affiliations:** 1 Department of Obstetrics and Gynecology, Center for Fetal Medicine, Karolinska University Hospital, Huddinge, Karolinska Institutet, Stockholm, Sweden; 2 Department of Medicine Solna, Rheumatology Unit, Karolinska University Hospital, Karolinska Institutet, Stockholm, Sweden; 3 Division for Clinical Immunology and Transfusion Medicine, Karolinska University Hospital, Huddinge, Karolinska Institutet, Stockholm, Sweden; 4 Department of Laboratory Medicine, Division of PathologySection of Perinatal Pathology, Karolinska University Hospital, Huddinge, Karolinska Institutet, Stockholm, Sweden; University of Cape Town, South Africa

## Abstract

**Background:**

Naturally acquired microchimerism may arise in the mother and her child during pregnancy when bidirectional trafficking of cells occurs through the placental barrier. The occurrence of maternal microchimerism (maternal cells in the offspring) has been associated with several autoimmune diseases, especially in children. Systemic Lupus erythematosus (SLE) is an autoimmune disorder with a resemblance to graft-versus-host disease. The aim of this study was to investigate the association between maternal microchimerism in the blood and SLE.

**Methodology/Principal Findings:**

Thirty-two patients with SLE, 17 healthy brothers of the patients, and an additional 12 unrelated healthy men were the subjects in this study. A single-nucleotide polymorphism unique to each mother was identified, and maternal microchimerism in the study group and in the control group was detected using a quantitative real-time polymerase chain reaction technique. No differences in the frequency or the concentration of maternal cells were apparent in the blood of patients with SLE or in that of the controls. Two patients and one control tested positive for maternal microchimerism, but the positive subjects were all negative at a follow-up 16 years later. The sensitivity of the method was estimated to 1/10.000.

**Conclusions/Significance:**

These results show no association between SLE and maternal microchimerism. The frequency of maternal microchimerism in the blood of adults overall may be lower than earlier reported.

## Introduction

Microchimerism (Mc) indicates the presence in an individual of a small number of cells from a genetically different person. The most common source of Mc is pregnancy: fetal cells pass the placental barrier, entering the mother and resulting in fetal microchimerism (FMc); conversely, maternal cells pass to her progeny and give rise to maternal microchimerism (MMc). Another source of chimerism is multiple pregnancies, when cells may pass from one fetus to another [1]. Mc could also result from iatrogenic interventions (i.e., blood transfusions, bone marrow and organ transplantations [2–4]).

Naturally acquired Mc may persist for a long time in both mother and progeny; Bianchi et al. showed that fetal cells may persist for as long as 27 years after birth in the blood of healthy mothers [5]. It has been reported that maternal cells in healthy individuals are also common in adults [6].

A common complication of haematopoietic stem-cell transplantation, a well-known chimeric state, is graft-versus-host disease (GvHD). Given that many autoimmune diseases bear a striking resemblance to GvHD, it has been hypothesized that autoimmune diseases may be related to the occurrence of chimerism. Because many autoimmune disorders predominantly affect women and because their incidence peaks during and after women’s child bearing years, it has been suggested that autoimmune diseases are associated with FMc. Some studies have reported an association between FMc and autoimmune disorders, whereas other have not found any, but it is notable that even in healthy controls, different levels of FMc in blood and tissue seem to be a rather common phenomenon [7–11].

So far, few studies have been made available on MMc and autoimmune disease. Maternal T cells have been described as engrafting in infants with severe combined immunodeficient disease [12,13]. A majority of them developed GvHD [13]. Other studies have suggested that MMc may play a role in the pathogenesis of juvenile dermatomyositis (JDM), juvenile idiopathic inflammatory myositis (JIIM), biliary atresia, neonatal lupus congenital heart block, type 1 diabetes, multiple sclerosis and systemic sclerosis [14–25].

MMc in humans has been reported to occur in fetuses, children, and adults, both in healthy offspring and in individuals who are born with a disease or develop one later in life [26–32]. The identified maternal cells in the progeny were composed of haematopoietic progenitor cells, T-cells, B-cells, monocytes/macrophages, NK-cells, and granulocytes [31,33,34].

Systemic lupus erythematosus (SLE) is an autoimmune disease characterized by the production of numerous autoantibodies that target structures located predominantly in the cell nucleus (antinuclear antibodies; ANA) or on cell membranes (antiphospholipid antibodies; APL). The antibodies form immune complexes that activate complement and lead to a state of systemic inflammation; simultaneous organ damage may occur at sites where these immune complexes are deposited (e.g., the kidneys [35]). The peak age of SLE incidence is between 30 and 40 years, and the female to male ratio in adults is approximately 9:1 [36].

Both environmental and genetic factors seem to contribute to the occurrence of SLE. Recent genome-wide association studies reveal that the strongest genetic influence resides in the MHC region on chromosome 6. The MHC class II alleles HLA-DRB1*03 and HLA-DRB1*15 are now well documented as risk alleles for SLE [37].

Previous studies investigating FMc in SLE have yielded divergent results. Mosca et al. were unable to find any difference between patients with SLE and healthy controls in terms of the incidence and the levels of fetal chimeric cells in blood, although the patients with a history of lupus nephritis had a higher number of fetal cells than patients with no such history did [38]. A study of renal tissue indicated that fetal cells were significantly more frequent in biopsies from the kidneys of SLE patients than in autopsy specimens without histomorphologic lesions. Some chimeric cells were identified as CD3+ and CD34+ [39]. The same group showed that organs from patients with SLE presented with chimerism more often than control organs did. The levels of chimerism corresponded to the degree of injury [40]. Johnson et al. compared organs affected by SLE with the unaffected organs of a woman who died from SLE; they found FMc in all the injured organs but not in the unaffected ones [41].

The association of MMc in cord blood with compatibility at a class II HLA locus between mothers and their fetuses, coupled with the finding that a disease with resemblance to SLE could be induced in mice when heterozygous progeny was transfused with homozygous parental lymphocytes, led Stevens et al. to investigate the HLA class II compatibility of male SLE patients and their mothers [30,42]. They concluded that this group had greater bidirectional HLA class II compatibility than controls did, suggesting that MMc could be more frequent in this group of patients [43].

The aim of this study was to investigate the association between MMc in blood and to compare adult patients diagnosed with SLE both to their healthy brothers and to unrelated healthy male adults.

## Materials and Methods

### Ethics statement

The Regional Ethics Committee, Stockholm, Sweden (2012/203–31/2) approved the study, and all participants provided informed written consent to participate.

The Local Ethics Committee at Karolinska University Hospital approved the original study in 1995 (95–178 (950616)). Written informed consent was obtained from all participants. The parents and caretakers of minors gave written consent on their behalf. The signed consent forms are stored with the research file.

### Participants and clinical specimens

Participating SLE patients were included in a genetic study at the Department of Rheumatology, Karolinska University Hospital, Stockholm, during the period 1995–1998. All included patients fulfilled at least four of the 1982 revised American College of Rheumatology Criteria for classification of SLE [44]. During the inclusion period, 208 patients were included in all. A rheumatologist interviewed and examined patients according to a structured protocol. Medical history was reviewed through interviews with the patients and an examination of medical records. SLE disease activity was determined using the Systemic Lupus Activity Measure (SLAM), and organ damage was measured using the Systemic Lupus International Collaborating Clinics (SLICC) damage index [45,46]. APS Antiphospholid syndrome (APS) was diagnosed according to the Sydney criteria [47]. First-degree relatives of SLE patients of Caucasian origin were asked to fill out a questionnaire, and blood samples were drawn for genetic and other analyses and stored at -700°C.

For our study, all female patients who reported in the questionnaire that they had never been pregnant and all the male patients were recruited. Thirty-two patients, with a mean age of 32 (range 18–52), fulfilled these criteria (30 women and 2 men) of the original 208.

The patients’ characteristics are shown in Table 1.

**Table 1:** Patients’ characteristics.

**Patient**	**Age at SLE onset**	**Age at inclusion**	**Gender**	**APS**	Discoid lupus*	Butterfly erythema*	Photo sensitivity*	Oral ulcers*	Arthritis*	Serositis*	Nephritis*	Haematology*	Neurology*	ANA*	Immunology*	Number of ACR criteria*	**SLAM**	**SLICC Damage index**
5	24	29	F	0	1	1	1	0	1	0	1	1	0	1	1	8	5	4
8	14	24	F	0	0	1	0	0	0	0	1	1	0	1	1	5	15	3
10	27	40	F	0	0	0	0	1	0	0	1	1	0	1	0	4	11	1
14	31	35	F	0	0	0	0	0	1	0	0	1	0	1	1	4	4	1
15	22	30	F	1	0	0	1	0	1	0	1	0	0	1	1	5	2	0
20	30	30	F	0	1	1	1	0	1	0	0	1	0	1	1	7	8	1
25	18	24	F	0	0	1	1	0	1	0	1	1	0	1	1	7	1	0
37	16	20	F	0	0	1	0	0	1	1	1	1	0	1	1	7	5	0
46	12	51	F	0	0	1	1	0	1	1	0	1	0	1	1	7	5	5
49	17	18	F	0	0	1	0	0	1	0	1	1	0	1	1	6	5	0
58	29	43	F	0	0	1	0	0	1	1	0	1	0	1	1	6	6	5
71	46	51	F	0	0	1	1	1	1	0	0	0	0	1	0	5	7	2
82	18	49	F	0	0	0	0	0	1	0	1	1	0	1	0	5	7	0
83	23	23	F	0	0	0	0	1	1	0	0	1	0	1	0	4	6	0
84	37	37	F	1	0	1	0	0	0	1	1	1	0	0	0	4	11	1
85	25	25	F	0	0	0	0	0	1	0	0	1	0	1	1	4	15	0
95	13	20	F	0	0	1	1	1	1	0	0	1	0	1	1	7	2	0
101	24	28	F	0	0	1	1	1	1	0	0	1	0	1	1	7	9	0
143	23	43	F	0	0	1	1	0	1	0	0	1	0	1	1	6	5	0
144	17	22	M	0	0	0	0	1	1	0	1	1	0	1	1	6	5	0
146	36	39	F	0	0	1	1	0	1	0	0	1	0	1	1	6	7	0
151	26	26	F	0	0	0	1	0	1	1	1	1	0	1	1	7	14	0
153	15	22	F	0	0	0	1	0	1	0	0	1	1	1	1	6	9	1
157	27	40	F	0	0	0	1	0	0	0	0	1	1	1	0	5	13	4
179	41	52	M	0	1	1	0	1	1	0	0	1	0	1	0	6	6	0
191	18	45	F	0	0	0	0	0	1	0	1	0	1	1	1	5	8	2
192	26	27	F	1	0	0	0	0	1	0	1	1	0	1	1	5	5	0
200	21	43	F	0	0	0	1	1	1	0	1	0	0	1	1	6	8	1
223	19	20	F	0	0	1	0	0	1	0	0	1	0	1	1	5	6	0
233	20	22	F	0	0	1	1	1	1	0	1	1	0	1	1	8	10	0
235	23	24	F	0	0	1	1	0	1	0	0	1	0	1	1	6	3	0
238	20	22	F	0	0	0	1	0	1	0	0	1	0	1	1	5	5	0

* Manifestations are defined according to American College of Rheumatology’s 1982 revised criteria for SLE [44], SLAM = Systemic Lupus Activity Measure, a validated measure of disease activity [45], SLICC = Systemic Lupus International Collaborating Clinics damage index, a validated cumulative organ damage [46], APS =antiphospholid syndrome was diagnosed according to the Sydney criteria [47]

The healthy control group comprised 17 brothers of the patients. All brothers of patients were included, even those whose patient-sisters were excluded from the study because of past pregnancy. In the questionnaire none of these brothers reported suffering from any chronic disease. The blood sampling of both patients and their brothers that was used for our study was conducted during the inclusion period of the genetic study (1995–1998). In addition, 12 healthy unrelated male individuals were recruited to compose the control group. The mean age of the 29 controls was 31 (range 10-54). There was no significant difference in age between the patients and controls (p-value = 0.8).

If they tested positive for MMc, patients and controls were evaluated for possible transfusions of blood products through a general registry on transfusion in Stockholm County, 1990–2012 (ProSang R).

### Quantitative real-time PCR

The methodology of chimerism analysis with real-time PCR has been described elsewhere [3]. In short, biallelic genetic systems were used to amplify maternal-specific alleles in SLE patients and in healthy controls. The allelic markers used for screening the mother and the child for differences in polymorhic regions of DNA were SO1a, SO2, SO3, SO4a, SO5b, SO7a, SO7b, SO8b, ID1, ID2, ID4 and ID7. If no differences were found the pair was screened for additional 10 markers (SO1b, SO4b, SO6, SO9b, SO10a, SO11b, ID9, ID10, and ID11and ID12). The markers are available at http://www.marshfieldclinic.org/research/genetics/QueryResults/searchSIDP.htm. For each biallelic system, one of the primers was from the polymorphic region to specifically amplify each allele, whereas the second primer and the probe were common to both alleles. An allele was considered informative when it was positive for maternal DNA and negative for the child’s DNA. Detection and quantification with one to three markers were performed with the 7500 Sequence Detection System (Applied Biosystems, Foster City, CA), using TaqMan technology. The amount of amplifiable DNA in each sample was assessed by parallel amplification of the reference gene glyceraldehyde phosphate dehydrogenase (GAPDH). All the samples were run in duplicate, and both maternal and patient/control DNA samples were included in each run. Relative quantification of recipient DNA was calculated according to the ΔΔCt method (Applied Biosystems, user bulletin 2), using GAPDH as a reference gene and the maternal DNA sample as a calibrator.

The amount of DNA used in the study varied between 150–450 ng. The specificity and sensitivity of the RQ-PCR method was determined for all markers included in this study using artificial DNA mixtures and varying DNA amounts. With 150-450 ng of DNA, a sensitivity of 0.01% was reached for most markers and no false positive results was found using 40 cycles of PCR amplification.

### Statistical Analysis

Statistical analyses for age differences between patients and controls were conducted with student’s t-test. Chi-square test was performed to analyze differences in MMc frequency between the two groups. Statistical analysis was performed using Statistica 10, StatSoft, Tulsa, USA.

## Results

The presence of MMc in the blood did not differ between SLE patients and healthy controls, 6% versus 3% (P-value = 0.65).

Maternal DNA was found in the blood of two of the 32 patients studied (patients 143 and 146); both were female. The concentration of maternal cells in their blood was 0.006% and 0.002%, respectively. Maternal DNA was found in two of the 32 patients studied, patients number 143 and 146. They were both female. The concentration of maternal cells was 0,006% and 0,002% respectively.

At inclusion patient 143 was 43 years old and patient 146 was 36 years. Disease duration was 20 and 3 years respectively. Both fulfilled 6 of the 1982 revised ACR criteria for SLE (median 6, range 4-8) [44]. Both were ANA positive and suffered from butterfly erythema, photosensitivity and arthritis. Patient 143 had an estimated SLAM disease activity score of 5 and patient 146 had a SLAM score of 7 (median 6, range 1-15) [45]. None of them had organ damage according to SLICC/ACR Damage index (median 0, range 0-5) [46]. In conclusion, their phenotype did not differ from the other patients.

One of the 29 healthy controls tested positive for the occurrence of MMc, with a concentration of 0.004%. He was 15 years old at the inclusion and the brother of patient 151.

After acquiring the results from this study, we contacted the three individuals who had tested positive for MMc and requested new blood samples in order to determine whether they were still carrying maternal DNA. We obtained samples from the two women with SLE and from the healthy control. After preparation of the DNA, and using the same primers and probes as before, we found that all three individuals now tested negative for MMc, 16 years after the first draw date.

One of the patients (patient 143) had received two blood transfusions, in 2001 and in 2002—that is, after the first blood test but before our follow-up.

## Discussion

This is to our knowledge the first study to investigate the correlation between maternal DNA in blood and SLE. The occurrence of maternal cells in blood among women with SLE was low and comparable to that among the control group. When studying Mc and its relation to a disease, it is an advantage to have healthy siblings as controls because of similarities in genetic constitution and environment.

The material in the present study is unique in the sense that none of the patients had a history of known pregnancy. This is important because in some cases of FMc, fetal DNA from an earlier pregnancy may carry the same polymorphic region as the mother used in the PCR reaction. Unrecognized pregnancies that end early in spontaneous abortions or terminated pregnancies may give rise to Mc in a woman, and such conditions may constitute significant confounding factors when studying both MMc and FMc [48,49].

The results of the present study show an overall low frequency for MMc compared to previous studies. We reviewed earlier published studies investigating MMc in the peripheral blood of healthy controls or in healthy study groups (Table 2). The lower frequency of MMc found in this study could be due to sensitivity of the method used. A sensitivity level of 0.01% may not be sufficient to detect low amount of MMc. Indeed, studies showing highest frequency of MMc have used methods with a sensitivity of at least 1/100.000. The methods used differ in detection rates and sensitivity. However, it is remarkable that among studies of adults, only one includes a defined group of male controls who all tested negative for MMc [24]. As mentioned above, early pregnancies that result in miscarriage or are aborted on request are known to give rise to FMc. Of the studies reviewed in Table 2, all but two studies of adults included only females in the study group and the control group. HLA genotyping was usually conducted for all children of the probands in order to ensure that testing for a noninherited, nonshared maternal HLA allele could not be confounded by a similar HLA allele from FMc. One study involved a mixed control group consisting of both men and women, but the frequency of MMc by gender was not reported separately [6]. Loubiere et al. showed that maternal cells were found in at least one subset of cells in 10 of 22 (45%) healthy parous women, compared to 2 of 9 (22%) nulliparous [33]. Although the cases were few, the researchers noted that the number of prior births tended to be higher among women with MMc than among those without.

**Table 2:** MMc in controls and healthy individuals in previously published reports.

**Children**	**Disease**	**Frequency of MMc in healthy controls**	**Mean age**	**Gender**	**Method**	**Sensitivity**	**Ref**
	Respiratory obstruction	4/20 (20%)	5	mixed	QRT-PCR**(SNP)***	1/10.000	50
	JDM	Healthy siblings: 12/39 (30%), other controls: 5/29 (17%)	10/20	boys/ boys	FISH	1/1000	14
		Healthy siblings: 11/48 (23%), other controls: 5/29 (17%)	10/20	mixed/ boys	PCR (HLA polymorphism)	1/50.000-100.000	
	JDM	5/35 (14%)	12	mixed	PCR (HLA polymorphism)	NP*	15
		Healthy siblings: 5/17 (29%), other controls: 2/10 (20%)	NP*	boys	FISH	NP*	
	Inflammatory bowel disease	2/15 (20%)	12	mixed	QRT-PCR **(HLA polymorphism)	1/25.000	55
	Type 1 Diabetes	Healthy siblings: 18/54 (33%), other controls: 4/24 (17%)	14/14	mixed/mixed	QRT-PCR** (HLA polymorphism)	1/20.000	23
	JIIM	0/9 (0%)	15	boys	FISH	1/10.000	17
	JIIM	2/21 (10%)	17	mixed	PCR (HLA polymorphism)	1/10.000	18
**Adults**	SSc	NP*(55%)	25	mixed	PCR (HLA polymorphism)	1/100.000-500.000	6
		0/2 (0%)	NP*	men	FISH		
	Healthy	12/31 (39%)	39	women	QRT-PCR** (HLA polymorphism)	1/20.000	33
	SSc	9/41 (22%)	40	women	QRT-PCR** (HLA polymorphism)	1/20.000	25
	Multiple sclerosis	0/22 (0%)	NP*	men	ARMS-PCR****	1/100.000	24
		0/38 (0%)	NP*	women	ARMS-PCR****	1/100.000	
	Healthy	6/15 (40%)	NP*	women	QRT-PCR** (HLA polymorphism)	1/100.000	34

* Not provided; ** Quantitative real-time PCR; *** single-nucleotide polymorphism; **** Amplification refractory mutation detection system polymerase chain reaction

For obvious reasons, genotyping of a past pregnancy that ended in termination or spontaneous abortion is not possible, leaving a certain amount of uncertainty regarding the source of Mc being studied. Thus, it may be that pregnancy is a major confounding variable in studies on MMc that so far has not received adequate attention. Caution is therefore recommended when studying the presence of MMc in relation to different phenotypes in adult women. Men and children will probably provide more accurate information regarding the occurrence of MMc.

We have previously studied the frequency of MMc in blood in children employing the technique used in the present study [50]; in children we found MMc in 20% of the sample, compared to 6% in the present study. Although the number of subjects studied was relatively low, the results indicate that MMc is a more common phenomenon in children. The dynamics of MMc with respect to time are not known, especially not in healthy subjects, but it is interesting that the patients and the control in the present study who tested positive for MMc tested negative 16 years later. The findings of a study by Gammill et al.—namely, that MMc decreased in prevalence and concentration with increasing parity—could reflect an impact of new grafts (FMc) but may also illustrate that MMc declines over time, since women’s age usually correlates with increasing parity [51].

The immunological status of both the healthy and the unhealthy individuals, as well as environmental factors, may affect changes in the levels and prevalence of maternal cells and also in immune modulatory treatment [18,52,53].

Maternal cells have been identified in multiple tissues, such as muscle, liver, heart, spleen, thymus, pancreas, and skin, both in individuals with autoimmune diseases and in others [14,19,22,23,31,54]. The chimeric maternal cells also seem to differentiate into organ-specific cells, indicating cellular plasticity [22,23,54]. In the present study we investigated only blood with regard to MMc; the situation could be different in tissues both in quantity and frequency, as it was in a patient with scleroderma who tested negative for maternal cells in blood but was found post mortem to harbour MMc in multiple tissues, including bone marrow [25]. Our recent study of MMc in children’s blood and tonsils and adenoids revealed an almost doubled concentration of maternal cells in the tissues compared to that in the blood, although all the children who tested positive in the blood were also positive in the tissues [50]. Suskind et al. did not find a significant relation between MMc and inflammatory bowel disease (IBD), but the frequency and quantity of MMc in tissues was higher, both in IBD patients and controls, than it was in the blood [55]. In this context, it might be more suitable to investigate Mc in affected tissues, where chimeric cells may exert their function.

To summarize, the present study did not show any association between MMc and SLE. The overall frequency of MMc in patients and controls was lower than that reported in previous studies, indicating that MMc might not be as common in adults as previously claimed—although differences in the sensitivity of methods must be taken into account. In studies on MMc and FMc, the pregnancy history of study subjects and the choice of control group might be of crucial importance. Although MMc may have implications for some autoimmune diseases, especially in children, in the present study we could not substantiate this relationship in adult SLE patients.
